# Availability, pricing, and affordability of essential medicines for pediatric population in Malawi

**DOI:** 10.3389/fphar.2024.1379250

**Published:** 2024-04-11

**Authors:** Francis Kachidza Chiumia, Cynthia Chithope-Mwale, Felix Abikoloni, Vanessa Matchaya, Tadala Gaviyawo, Felix Khuluza

**Affiliations:** Department of Pharmacy, School of Life Sciences and Allied Health Professions, Kamuzu University of Health Sciences, Blantyre, Malawi

**Keywords:** availability and affordability, Malawi, pediatric formulations, children, medicine pricing

## Abstract

**Objective:**

Lack of access to essential medicines negatively impacts on the quality of healthcare delivery and increases morbidity and mortality, especially to the vulnerable pediatric population. We assessed the availability, pricing, and affordability of pediatric formulations in Malawi.

**Methodology:**

The study was conducted in 76 health facilities (public, faith-based and private pharmacies, and clinics) from the northern and southern regions of Malawi from March to May 2023. We adapted the WHO/HAI method for the assessment of both availability and pricing of medicines. Data on availability were collected from stock card records using a WHO/HAI template and medicine prices were provided by the pharmacy personnel who were managing the facilities. Availability of medicines was calculated as the percentage of facilities which had a stock of the respective medicine at the time of data collection while medicine prices was assessed by calculating the median prices of each medicine. To assess the affordability of the medicines, we calculated the number of days it takes for a person who is receiving the government-set minimum wage to work to pay for a treatment course of common indications. The study was approved by the KUHES ethics committee under the numbers U.12/22/3900 and U.12/22/3903.

**Results and conclusion:**

The overall availability of pediatric medicines was 38.1% for public health facilities, 53.7% for private retail pharmacies and drug stores, 49.5% for private clinics and 48.3% for Christian Health Association of Malawi (CHAM) facilities. We found the illegal availability of prescription-only medicines of up to 54% in medicine stores. Medicine median prices were higher in the private clinics followed by retail pharmacies and drugs stores. CHAM had the lowest median prices for medicines of all the sectors. More than 50% of medicines were found to be affordable as less than a day’s wage was required to purchase the treatment. We found poor availability of pediatric formulation among public, CHAM, and private sectors in Malawi. This may affect the quality of care among pediatric patients and therefore contribute to morbidity and mortality in Malawi. The supply of medicines and health commodities needs to consider needs of special populations such as children to achieve universal health coverage.

## Introduction

Lack of access to essential medicines negatively impacts the quality of healthcare delivery and increases morbidity and mortality (Toroitich et al., 2022). In 2017, the World Health Organization estimated that around two billion people globally had no access to essential medicines (World Health Organization, 2017). This is a barrier to achieving universal health coverage and undermines the right to access healthcare (Perehudoff, 2020). Although effective measures have been taken to improve access to essential medicines in some countries, Low-Middle Income Countries (LMICs) face significant challenges to overcome this problem due to poor health system infrastructure, dependence on imported medicines, procurement and supply chain constraints, inadequate health insurance and lack of human and financial resources (Babigumira et al., 2017; Ooms et al., 2023; Yenet et al., 2023). Vulnerable populations including children continue to be prone to facing heavy consequences of medicine inaccessibility as some of the few available medicines may not be of acceptable formulation suitable for their clinical needs (Bigdeli et al., 2013).

Morbidity and mortality rates are higher among the pediatric population than other age groups globally (World Health Organization, 2017; Karlsson et al., 2022). In 2022, under five mortality rate (U5MR) was at 37 per 1,000 live births globally (Unicef, 2023). Whilst over the past years U5MR has significantly improved across high-middle income countries, it remains high in LMICs. In 2015, the Sub-Saharan African region contributed to almost 50% of the global death among under-five children and U5MR remained as high as 73 death per 1,000 live births as of 2017 (Exemplars_in_Global_Health, 2023; Droti et al., 2019). Preventable and treatable infections such as pneumonia, diarrhea and malaria are among the leading cause of death among the pediatric population (World Health Organization, 2023a). Improving the quality of healthcare delivery may therefore significantly reduce morbidity and mortality among pediatric patients.

To counter the problems arising from poor availability, affordability and acceptability of essential medicines for children, the WHO launched a campaign named “Make Medicines Child Size” in 2007 and “Better Medicines for Children Project” in 2009 (Watts, 2007; Gitanjali, 2011). The campaign aimed at enhancing the availability and quality of pediatric formulations by promoting awareness and establishment of policies that are driven by research and informed regulatory actions (Watts, 2007; Yewale and Dharmapalan, 2012). Further, the first WHO model list of essential medicines for children (WHO EMLc) was published to provide guidance on the most important medicines that are required to be always available at a healthcare facility to meet the needs of the pediatric population while maximizing the cost effectiveness (Hoppu and Sri Ranganathan, 2015).

A study conducted in eight Sub-Saharan African countries by Droti et al. found low availability of essential medicines for pediatric patients. Primary healthcare facilities had 48% of the priority medicines while hospitals had 58% of the medicines (Droti et al., 2019). Another study in Western Ethiopia found availability of 22 essential medicines to be 43% and 42.8% for public and private sector respectively. Furthermore, the study found that the medicines were unaffordable as the lowest priced medicines were sold at 1.18 and 1.54 times their international reference price (IRP) in public and private sector respectively (Sado and Sufa, 2016). On the other hand, the availability of pediatric medicines in South Africa was estimated at 64.% and 84% in the public and private sector respectively (Mahadeo et al., 2022). This is not surprising, with South Africa being an upper-middle income country and an outlier within the region.

In 2017, a study done in Malawi assessed the availability of 50 essential medicines including few selected pediatric formulations such as paracetamol and amoxicillin suspension. Of the selected 12 public, 11 faith-based and 21 private facilities, the study found that almost 65% of the medicines were not available and were priced higher as compared to other countries (Khuluza and Haefele-Abah, 2019). However, another study found a high availability of up to 93% and 100% for public and faith-based facilities respectively for antimalarial medicines. The higher availability of antimalarial medicines in Malawi was attributed to support from international donors including the Global Fund and the United States of America President’s Malaria Initiative (Medicines for Malaria Venture, 2013). Despite the reported high morbidity and mortality rates among children (World Health Organization, 2023a), data on availability and pricing for pediatric formulations is limited in most countries including Malawi. Therefore, the potential effect of the accessibility of pediatric medicines on the delivery of healthcare among children remains unknown. In the present study, we assessed the prices, availability, and affordability of essential medicines for pediatric population in selected districts in Malawi.

## Methods

### Study design

This was a descriptive cross-sectional study design that assessed the availability, pricing, and affordability of 43 essential pediatric medicines in Malawi. Data were collected from public health facilities, Christian Health Association of Malawi (CHAM), private retail/community pharmacies and private clinics in selected districts across the country. We used the methodology developed by the World Health Organization (WHO) and Health Action International (HAI) on measuring medicine prices, availability and affordability in the development of the study design, data collection and analysis (World-Health-Organization(WHO), 2012). Data was collected from March to May 2023.

### Study sites and sampling procedures

The study was conducted in five districts, three of which are in the northern region of Malawi (Mzimba, Nkhata-Bay and Rumphi) and the other two in the southern region (Zomba and Blantyre districts). Since the last study on availability and affordability of medicines (Khuluza and Haefele-Abah, 2019), there has been increased private health facilities being opened in the northern region, hence the inclusion of the same. We obtained a list of registered facilities from the Ministry of Health (public and CHAM facilities), Pharmacy and Medicines Regulatory Authority (retail pharmacies) and Medical Council of Malawi (for private clinics). We randomly pre-selected a total of 90 registered health facilities within the study districts, exceeding the minimum number of sites of 50 from the WHO/HAI methodology (World-Health-Organization(WHO), 2012).

**FIGURE 1:**
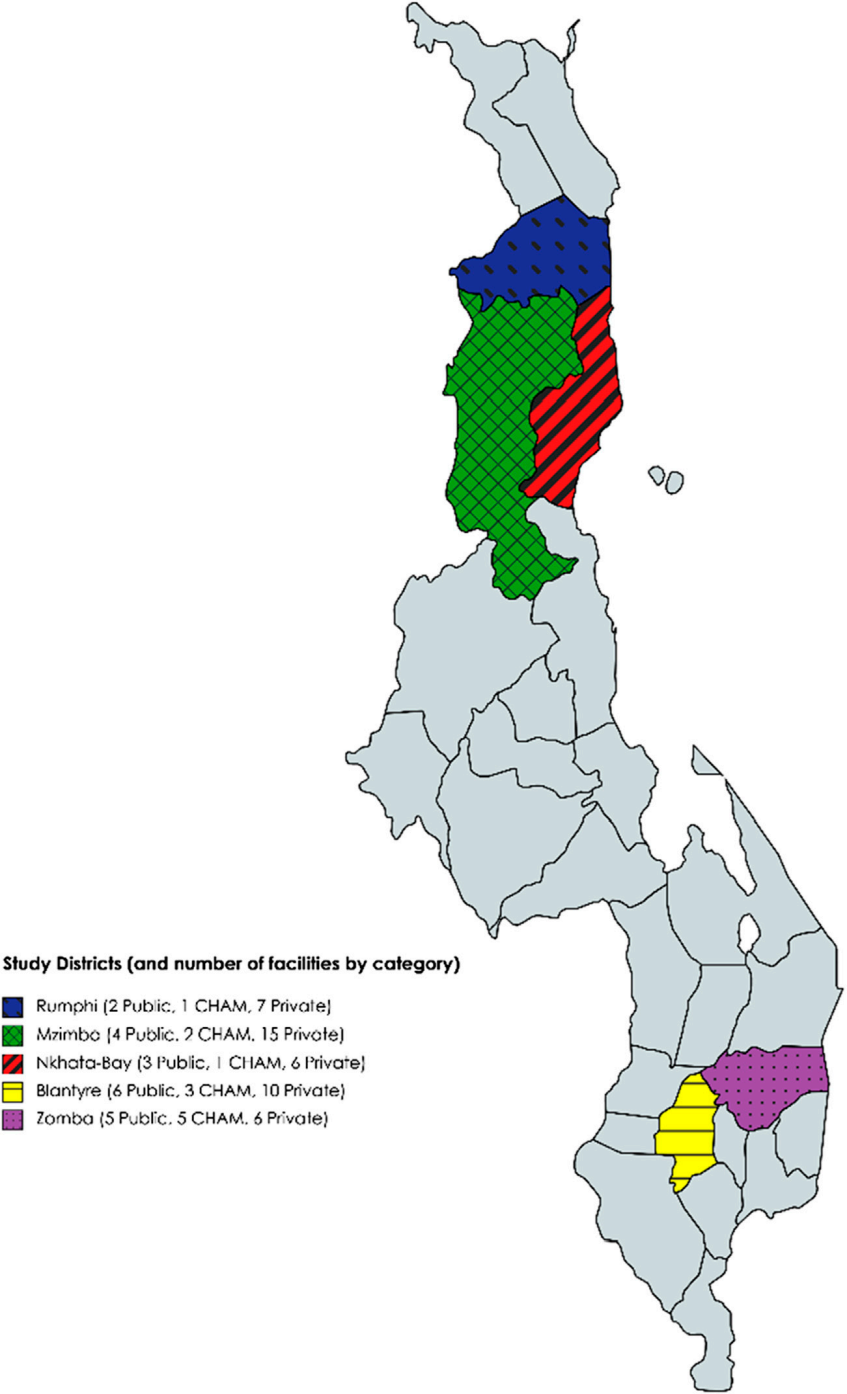
Map of Malawi showing study districts.

Of the 90 randomly selected facilities, we collected data from 19 retail pharmacies and 11 medicine stores (giving a total of 30 in this sector), 20 public health facilities, 12 CHAM facilities and 14 private clinics giving us a total of 76 sites (Figure 1 showing distribution of study sites). The reduced number of study sites was as a result of some facilities (9 private/CHAM) declining to provide any data despite repeated visits to the sites even though confidentiality to the data was guaranteed. The other five sites could not be located, which could be as a result of closing their businesses. Despite having some challenges in some facilities, the response rate for the study was very good (84.4%) and above most studies (Fincham, 2008). The public health facilities included three central hospitals, five district hospital pharmacies, and 12 primary health centers.

### Criteria for selection of medicines

We used Malawi Standard Treatment Guidelines (MSTG) 2015 that incorporated Malawi Essential Medicines List (MEML) as a main backbone in selecting pediatric medicines for the study (Ministry-of-Health, 2023). This was supplemented with information from World Health Organization Essential Medicines List for Children and Malawi Pediatric Handbook (World Health Organization, 2021). This resulted in the initial selection of 50 medicines for the study. However, during data collection, we realized that seven of the initially selected products are not stocked in any facility and as such were removed from data analysis. Table 1 is the list of the medicines that were included in the study while Supplementary Table S1 contains the details on why each medicine was included in the study.

**TABLE 1:** Availability of essential pediatric medicines in different types of health facilities in Southern and Northern Malawi in 2023.

	Medicine name	MEML^1^	ATC CODE^2^	Public-district hospital and health centers (n = 20) (%)	Private pharmacies and drug stores (n = 30) (%)	Private clinics (n = 14) (%)	CHAM^c^ (n = 12) (%)
1	Acyclovir 200 mg tablet	HVA	J05AB01	85.0	63.3	57.1	58.3
2	Albendazole 200 mg tab	HEA	P02CA03	40.0	76.7	50.0	58.3
3	Albendazole suspension 200mg/5 mL	NA	P02CA03	0.0	60.0	14.3	0.0
4	Aminophylline 100 mg tab	HVA	R03DA05	85.0	70.0	85.7	75.0
5	Amoxicillin 250 mg dispersible tab	HVA	J01CA04	70.0	70.0	92.9	66.7
6	Amoxicillin suspension 125mg/5 mL	HVA	J01CA04	15.0	56.7	92.9	83.3
7	Artemether/Lumefantrine suspension	NA	P01BF01	0.0	6.7	7.1	0.0
8	Artesunate IV 60 mg ampoule	CVB	P01BE03	100.0	20.0	50.0	100.0
9	Artesunate suppository 100 mg	HVA	P01BE03	40.0	0.0	0.0	50.0
10	Azithromycin 250 mg tab	DEA	J01FA10	35.0	53.3	28.6	33.3
11	Azithromycin suspension 200mg/5 mL	DEA	J01FA10	40.0	36.7	28.6	0.0
12	Benzyl penicillin Sodium, 5MU vial	HVA	J01CE01	70.0	43.3	85.7	100.0
13	Carbamazepine 200 mg tab	DEB	N03AF01	40.0	63.3	28.6	33.3
14	Clindamycin 150 mg cap	DEB	J01FF01	0.0	3.3	0.0	8.3
15	Co-amoxiclav 625 mg tab	CEA	J01CR02	10.0	60.0	42.9	8.3
16	Co-amoxiclav suspension 156mg/5 mL	NA	J01CR02	0.0	40.0	28.6	16.7
17	Cotrimoxazole 120 mg tab	NA	J01EE01	75.0	36.7	42.9	75.0
18	Cotrimoxazole suspension 240mg/5 mL	NA	J01EE01	0.0	53.3	21.4	50.0
19	Erythromycin 250 mg tab	HVA	J01FA01	85.0	60.0	42.9	58.3
20	Erythromycin suspension 125mg/5 mL	DVA	J01FA01	20.0	53.3	57.1	58.3
21	Ferrous sulphate 200 mg tab	HVA	B03AD03	25.0	50.0	35.7	50.0
22	Ferrous sulphate suspension 60mg/5 mL	HEA	B03AA07	0.0	0.0	0.0	8.3
23	Flucloxacillin 250 mg cap	DVA	J01CF05	60.0	43.3	21.4	25.0
24	Flucloxacillin suspension 125mg/5 mL	DVA	J01CF05	10.0	10.0	7.1	0.0
25	Gentamicin 80mg/2 mL ampoule	HVA	J01GB03	60.0	66.7	92.9	91.7
26	Ibuprofen 200 mg tab	DEA	M01AE01	15.0	90.0	71.4	75.0
27	Ibuprofen suspension 100mg/5 mL	NA	M01AE01	5.0	40.0	28.6	8.3
28	Ketoconazole 200 mg tab	DVA	J02AB02	10.0	63.3	64.3	41.7
29	Mebendazole 500 mg tab	HEA	P02CA01	0.0	40.0	0.0	0.0
30	Metronidazole 200 mg tab	HVA	J01XD01	75.0	66.7	92.9	100.0
31	Metronidazole suspension 200mg/5 mL	DVA	J01XD01	25.0	70.0	64.3	58.3
32	Nalidixic acid 500 mg tab	DVA	J01MB02	0.0	10.0	0.0	0.0
33	Nystatin oral suspension 100,000IU/mL	NA	D01AA01	75.0	73.3	71.4	66.7
34	Oral rehydration salts	HVA	A07CA	90.0	80.0	92.9	91.7
35	Paracetamol 500 mg tab	HVA	N02BE01	95.0	96.7	92.9	91.7
36	Paracetamol suspension	HVA	N02BE01	20.0	93.3	92.9	58.3
37	Phenobarbitone 30 mg tab	HVA	N03AA02	35.0	73.3	78.6	58.3
38	Praziquantel	HEA	P02BA01	30.0	53.3	0.0	25.0
39	Promethazine 25 mg tab	HVA	R06AD02	65.0	76.7	85.7	75.0
40	Promethazine hydrochloride 5mg/5 mL syrup	DEA	RO6AD02	0.0	60.0	35.7	16.7
41	Salbutamol 4 mg tab	HVA	R03AC02	50.0	76.7	100.0	91.7
42	Salbutamol suspension 2mg/5 mL	NA	R03AC02	5.0	73.3	42.9	41.7
43	Zinc sulphate dispersible 20 mg tab	HVA	A12CB01	80.0	76.7	100.0	66.7
	Overall availability			38.1	53.7	49.5	48.3

^a^ MEML = Malawi Essential Medicines List. The EML, categorized medicines according to the level of facility at which they are found (H=Health Centre, D = district hospital, C=Central Hospital), therapeutic importance of the medicines (V = vital medicines which are used to treat life threatening illnesses; E = Essential medicines which are used to treat common but less severe illnesses) and the procurement system for the medicines (A = medicines procured almost monthly to cover needs of huge proportion of the population; and B = medicines required for fewer number of patients and therefore not procured by monthly routines).

^b^ ATC, anatomical, Chemical and Therapeutic Classification (As classified by World Health Organization.

^c^ CHAM, christian health association of malawi.

### Data collection, calculation of medicine availability and prices

Two trained teams of pairs (CCM and VM; and FA &TG) each were assigned to collect the data. We used WHO/HAI template for data collection with minor modification that was in line with previous studies (Khuluza and Haefele-Abah, 2019; World-Health-Organization(WHO), 2012). The modification concerned collecting prices regardless of branding of the medicines. This was done as Malawi has very few branded products on the market as such it was unlikely to collect three prices for each medicine (branded, most sold generic and lowest priced). The modified data collection form has been submitted as Supplementary Table S2. We recorded the medicine availability and price as provided by the responsible personnel, mainly the person in charge of the pharmacy (for public health facilities, CHAM, and retail pharmacies). While for private clinics, information was mainly provided by the owners of the facilities who in most cases were clinicians or nurses. Public health facilities provided availability information only as they provide medicines for free (Khuluza and Haefele-Abah, 2019).

We calculated medicine availability as the percentage of facilities which had stock of the respective medicine at the time of the visit, irrespective of the amount and pack size available, as recommended by the WHO/HAI methodology. The availability of medicines was compared among the different healthcare sectors: public, CHAM (faith-based) and private sectors. In terms of level of care, we conducted a sub-analysis on the availability of medicines that are supposed to be available in primary level facilities. All descriptive analyses were done in Microsoft Excel. We used 80% as a minimum target for assessing medicine availability in this study, that is in line with WHO, Tadesse T et al. and previous study in Malawi (World Health Organization, 2013; Bazargani et al., 2014; Khuluza and Haefele-Abah, 2019).

We collected medicine prices in local currency (Malawi Kwacha, MWK) that were converted to US $ using the average Reserve Bank of Malawi exchange rate for the month of April 2023 (1 US $ = 1036.25 MWK), in which the data were collected (Reserve_Bank_of_Malawi, 2023). The conversion to US Dollar allows international comparison of prices during the time of study as Malawian currency has been unstable since 2021. For example, the exchange rate of 1 USD was 1036.25 during the time of data collection (April 2023), but was subsequently devalued that by December of 2023, when 1 USD was equivalent to 1868.25 MWK (https://www.exchangerates.org.uk/USD-MWK-exchange-rate-history.html). For facilities where there was more than one price per product, we selected the cheapest one for inclusion in the data analysis. The lack of availability of originator brands, and thus lack of categorization, did not have an adverse impact on the results of the study. We also did a sub-analysis of medicine availability based on the level of health facility where the medicine should be available, as well as disaggregated data for private drug stores and retail pharmacies.

### Calculation of courses of treatment and medicine affordability

A total of 43 medicines were assessed for affordability based on the treatment regimens for common pediatric conditions in Malawi (Ministry-of-Health, 2023; IHMA, 2023). We used the Malawi Standard Treatment Guidelines (MSTG) 2015 supplemented by pediatric handbook for Malawi to calculate the amount of dosage units (bottles/tablets/capsules/vials) required for one course of treatment or for a monthly treatment in case of chronic conditions (Ministry-of-Health, 2023; Phillips et al., 2008). The majority of pediatric treatment are based on the weight of the child. In this study, we used a weight range of 15–20 kg in the calculation of the treatments. We multiplied the weights by the dosage per kilogram in order to find the course of treatment. The course of treatment was then converted to cost by multiplying it with the median price of a unit of medicine. A unit of medicine was the lowest unit that can be administered to the patient, which was either a tablet or capsule or a milliliter of medicine in cases of syrups. The total cost of the treatment was then divided by the minimum wage of Malawi to establish the number of days it would require for the worker to afford the treatment. Among other conditions, we included both complicated and uncomplicated malaria, sepsis, diarrhea, upper and lower respiratory tract infections including pneumonia, meningitis, seizures, and anemia. Supplementary Table S3) indicates the diseases condition that was considered for estimating the affordability of each medicine that was included in the study. For benzyl penicillin and gentamicin injections, a treatment duration of 3 days (before changing to oral medication) was chosen as stated in the treatment guidelines. For oral suspension or solutions, a bottle of 100 mL was used as a complete dose for the age range 0–2 years, and two bottles above 2 years while for any child above 10 years, we used available oral solid dosage forms (tablets or capsules). The daily minimum wage of the lowest-paid unskilled worker, as legislated by the Malawian government, was used to measure local affordability and the number of days’ wages needed to purchase a course of treatment (1923.08 MWK = 1.86 US$) (Wage_Indicator, 2023).

### Ethics approval and consent to participate

The Institution Review Board of Kamuzu University of Health Sciences-Malawi (College of Medicine Research and Ethics Committee (COMREC) under study numbers U.12/22/3900 and U.12/22/3903) granted approval. In addition, permission was sought from the hospital director or directorates of health and social services in Zomba, Blantyre, Mzimba, Rumphi and Nkhatabay before data collection. The study did not collect any patient data. The study was approved in line with the principles of the declaration of Helsinki.

## Results

### Availability of pediatric medicines

We here present data for 43 medicines that were analyzed. The overall availability of pediatric medicines in all sectors was 38.1% for public health facilities, 53.7% for private retail pharmacies and drug stores, 49.5% for private clinics and 48.3% for CHAM facilities. Nalidixic acid, mebendazole and ferrous sulphate syrup had the lowest availability with zero availability in three of the four sectors. Artesunate injection had 100% availability in CHAM and public facilities, however its availability in private pharmacies and private clinics was very low. Artemether/Lumefantrine suspension had zero availability in CHAM and public facilities, with poor availability in private sectors (6.7% and 7.1% in private pharmacies and clinics respectively) Table 1.

**FIGURE 2:**
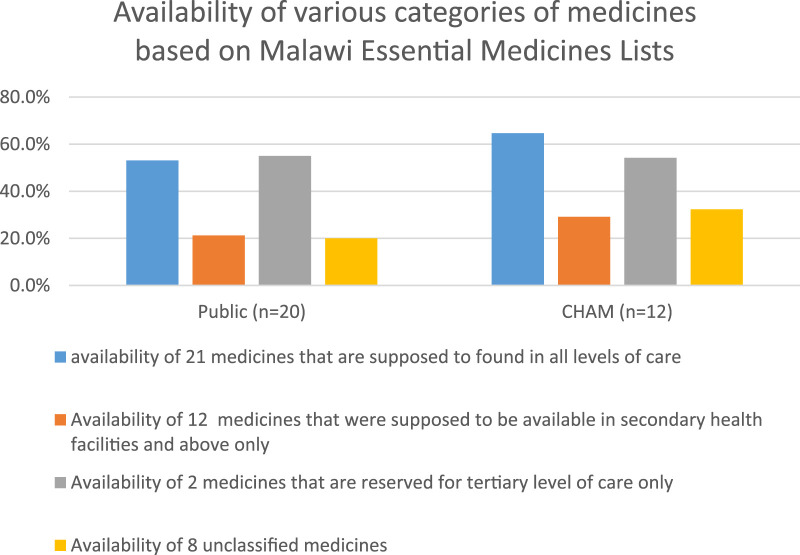
Graph showing availability of medicines based on Malawi Essential Medicines Lists categorization.

We further analyzed the availability of medicines based on the level of health facility where they were supposed to be found. This was done in order to assess the adherence of the public sector to Malawi Essential Medicines List in their distribution of medicines. This analysis included CHAM and public health facilities only as in most cases CHAM facilities are at the level of primary care which is similar to most of the public facilities where the data were collected. A sub-analysis of 21 medicines that are supposed to be found in health centers (primary healthcare facilities) and throughout the system showed a higher medicines availability at 53.1% in public facilities, and 64.7% for CHAM facilities. While for those medicines that were not supposed to be at a health center (primary-level care) revealed a below 50% availability, Figure 2. Surprisingly, availability of artesunate injectable was 100% in public sector and CHAM, while amoxicillin plus clavulanic acid (a tertiary level medicine) was also available in some health centers, Table 2. In addition, there was presence of unclassified medicines in both CHAM and public health facilities, Table 2. Unclassified medicines are the ones that are not appearing in Malawi’s Essential Medicines List of both 2015 and 2023.

**TABLE 2:** Shows the availability of unclassified medicines in the public and CHAM sectors.

Medicine name	MEML^***^	Public health facilities (n = 20)	CHAM (n = 12)
Albendazole suspension 200mg/5 mL	NA	0.00%	0.00%
Artemether/Lumefantrine suspension	NA	0.00%	0.00%
Co-amoxiclav suspension 156mg/5 mL	NA	0.00%	16.70%
Cotrimoxazole 120 mg tab	NA	75.00%	75.00%
Cotrimoxazole suspension 240mg/5 mL	NA	0.00%	50.00%
Ibuprofen suspension 100mg/5 mL	NA	5.00%	8.30%
Nystatin oral suspension 100,000IU/mL	NA	75.00%	66.70%
Salbutamol suspension 2mg/5 mL	NA	5.00%	41.70%
Overall availability		20.0%	32.3%

***NA = not applicable: these medicines are not found in Malawi Essential Medicines List, even though they are internationally recommended to be available for pediatric population.

### Pediatric medicine median prices

Pediatric medicine median prices were higher in the private clinics followed by retail pharmacies and drugs stores. CHAM had the lowest median prices of all the sectors. It should be noted that the public health sector in Malawi provides their services free of charge including medicines, as such there was no such comparison on prices in the public sector. In addition, certain categories of medicines like first line antimalarial medicines, are provided free of charge also in the CHAM sector, as such there was no comparison of their median prices. There was a wide variation in the median prices of certain medicines like albendazole among the sectors, with private retail pharmacies charging four times the price in the CHAM while private clinics charged six times the price in the CHAM, Table 3. The median price for paracetamol tablet was the same across all the sectors. Amoxicillin suspension, albendazole and metronidazole tablets had the lowest variation in median prices among the sectors (CHAM, Private Pharmacies. and Clinics).

**TABLE 3:** Median prices of various medicines among the various health facilities.

Medicine name	MEML	ATC CODE	Retail pharmacy		Private clinics		Christian health Association of Malawi (CHAM)	
			Median price in MWK	Median price in US cents	Median price in MWK	Median price in US cents	Median price in MWK	Median price in US cents
Acyclovir 200 mg tablet	HVA	J05AB01	115	11.10	190	18.34	60.00	5.79
Albendazole 200 mg tab	HEA	P02CA03	400	38.60	600	57.90	100.00	9.65
Albendazole suspension 200mg/5 mL	NA	P02CA03	63.5	6.13	NA^*^	NA^*^	NA^*^	NA^*^
Aminophylline 100 mg tab	HVA	R03DA05	17	1.64	30	2.90	30.00	2.90
Amoxicillin 250 mg dispersible tab	HVA	J01CA04	67	6.47	80	7.72	50.00	4.83
Amoxicillin suspension 125mg/5 mL	HVA	J01CA04	18	1.74	20	1.93	15.00	1.45
Artesunate IV 60 mg ampoule	CVB	P01BE03	3100	299.16	4,000	386.01	NA^*^	NA^*^
Azithromycin 250 mg tab	DEA	J01FA10	450	43.43	NA^*^	NA^*^	563.63	54.39
Azithromycin suspension 200mg/5 mL	DEA	J01FA10	166.6667	16.08	200	19.30	NA^*^	NA^*^
Benzyl penicillin Sodium, 5MU vial	HVA	J01CE01	1080	104.22	2000	193.00	950.00	91.68
Carbamazepine 200 mg tab	DEB	N03AF01	100	9.65	77.5	7.48	NA^*^	NA^*^
Co-amoxiclav 625 mg tab	CEA	J01CR02	376.5	36.33	550	53.08	NA^*^	NA^*^
Co-amoxiclav suspension 156mg/5 mL	NA	J01CR02	39.25	3.79	45.75	4.41	NA^*^	NA^*^
Cotrimoxazole 120 mg tab	NA	J01EE01	35	3.38	62.5	6.03	NA^*^	NA^*^
Cotrimoxazole suspension 240mg/5 mL	NA	J01EE01	13.75	1.33	24	2.32	11.82	1.14
Erythromycin 250 mg tab	HVA	J01FA01	112.5	10.86	142.5	13.75	71.83	6.93
Erythromycin suspension 125mg/5 mL	DVA	J01FA01	21.5	2.07	26.5	2.56	15.00	1.45
Ferrous sulphate 200 mg tab	HVA	B03AD03	20	1.93	40	3.86	40.00	3.86
Flucloxacillin 250 mg cap	DVA	J01CF05	146	14.09	NA^*^	NA^*^	NA^*^	NA^*^
Gentamicin 80mg/2 mL ampoule	HVA	J01GB03	337.5	32.57	500	48.25	300.00	28.95
Ibuprofen 200 mg tab	DEA	M01AE01	24	2.32	35	3.38	40.00	3.86
Ibuprofen suspension 100mg/5 mL	NA	M01AE01	13	1.25	19.5	1.88	NA^*^	NA^*^
Ketoconazole 200 mg tab	DVA	J02AB02	100	9.65	150	14.48	100.00	9.65
Mebendazole 500 mg tab	HEA	P02CA01	91.665	8.85	NA^*^	NA^*^	NA^*^	NA^*^
Metronidazole 200 mg tab	HVA	J01XD01	21.965	2.12	50	4.83	30.00	2.90
Metronidazole suspension 200mg/5 mL	HVA	J01XD01	15	1.45	20	1.93	12.75	1.23
Nystatin oral suspension 100,000IU/mL	HVA	D01AA01	55.5	5.36	73.665	7.11	58.34	5.63
Oral rehydration salts	HVA	A07CA	850	82.03	600	57.90	400.00	38.60
Paracetamol 500 mg tab	HVA	N02BE01	30	2.90	30	2.90	30.00	2.90
Paracetamol suspension	HVA	N02BE01	12.75	1.23	15	1.45	15.00	1.45
Phenobarbitone 30 mg tab	HVA	N03AA02	17	1.64	30	2.90	37.50	3.62
Praziquantel	HEA	P02BA01	500	48.25	NA^*^	NA^*^	NA^*^	NA^*^
Promethazine 25 mg tab	HVA	R06AD02	30	2.90	37.5	3.62	30.00	2.90
Promethazine hydrochloride 5mg/5 mL syrup	DEA	R06AD02	15	1.45	25	2.41	NA^*^	NA^*^
Salbutamol 4 mg tab	HVA	R03AC02	13.333	1.29	28.335	2.73	30.00	2.90
Salbutamol suspension 2mg/5 mL	NA	R03AC02	10	0.97	15	1.45	7.50	0.72
Zinc sulphate dispersible 20 mg tab	HVA	A12CB01	50	4.83	65	6.27	39.27	3.79

^*^There was no or insufficient data to calculate the median prices.

### Comparison of medicine availability and prices between private pharmacies and drug stores

For private retail pharmacies and drug stores, prescription only medicines (POM) are supposed to be found in retail pharmacies only and not drug/medicine stores. Drug stores are supposed to keep Pharmacy Only (P) and General Sales List (GSL) medicines category as mandated by the Pharmacy and Medicines Regulatory Authority (PMRA). However, a sub-analysis of 19 private retail pharmacies and 11 drug stores observed widespread availability of POM medicines in the drug/medicine stores. Overall availability of medicines in the retail pharmacies was 64.8% while the drug stores was 42.7%. We observed widespread availability of POM medicines in drug stores with as high as 54% for metronidazole suspension. Medicine median prices for retail pharmacies were lower than drug stores except for aminophylline tablet, Amoxicillin tablet/cap, benzyl penicillin and ORS (Table 4).

**TABLE 4:** Comparison of availability and median price of medicines in the Retail pharmacies and Drug/Medicine stores.

1USD =	1036.25				Median price in US cents		Medicine availability	
No.	Medicine name	Medicine Strength	Dosage form	Pharmacy and medicines regulatory authority category	Drug stores	Retail pharmacy	Drug stores	Retail pharmacies
1	Acyclovir	200 mg	Tab	POM	14.48	10.37	45.5%	73.7%
2	Albendazole	200 mg	Tab	P	43.43	38.55	90.9%	68.4%
3	Albendazole suspension	200mg/5 mL	oral suspension	P	7.72	6.13	54.6%	63.2%
4	Aminophylline	100 mg	Tab	POM	1.61	1.93	63.6%	73.7%
5	Amoxicillin dispersible tablet	250 mg	cap/tab	POM	6.47	6.59	45.5%	84.2%
6	Amoxicillin suspension	125mg/5 mL	suspension	POM	1.93	1.74	45.5%	63.2%
7	Artemether/Lumefantrine suspension	20mg/120 mg	Tab	P	4.05	4.54	9.1%	5.3%
8	Artesunate IV	60 mg	ampoule	POM	265.38	349.82	18.2%	21.1%
10	Azithromycin	250 mg	Tab	POM	64.37	38.60	27.3%	68.4%
11	Azithromycin suspension	200 mg/ml	oral suspension	POM	16.12	14.48	27.3%	42.1%
12	Benzyl penicillin Sodium	5000000 IU	IV	POM	96.50	110.01	27.3%	52.6%
13	Carbamazepine	200 mg	Tab	POM	14.48	9.65	27.3%	84.2%
15	Co-amoxiclav	625 mg	Tab	POM	38.60	34.51	18.2%	84.2%
16	Co-amoxiclav suspension	125mg/5 mL	oral suspension	POM	22.63	3.76	18.2%	52.6%
17	Cotrimoxazole	120 mg	Tab	POM	3.86	2.75	27.3%	42.1%
18	Cotrimoxazole suspension	48 mg/mL	oral suspension	POM	1.35	1.33	36.4%	63.2%
19	Erythromycin	250 mg	Tab	POM	10.86	10.86	27.3%	79.0%
20	Erythromycin suspension	125mg/5 mL	oral suspension	POM	1.93	2.12	27.3%	68.4%
21	Ferrous sulphate	200 mg	Tab	P	1.93	1.60	63.6%	42.1%
23	Flucloxacillin	250 mg	Cap	POM	12.06	14.09	18.2%	57.9%
24	Flucloxacillin suspension	125mg/5 mL		POM	1.16	2.41	9.1%	10.5%
25	Gentamicin	80mg/2 mL	IM	POM	38.60	30.88	27.3%	89.5%
26	Ibuprofen	200 mg	Tab	P	2.69	2.22	90.9%	89.5%
27	Ibuprofen suspension	125mg/5 mL	oral suspension	P	1.35	1.16	27.3%	47.4%
28	Ketoconazole	200 mg	Tab	POM	9.65	9.65	45.5%	73.7%
29	Mebendazole	100 mg	Tab	P	9.65	6.47	27.3%	47.4%
30	Metronidazole	200 mg	Tab	POM	2.28	1.96	36.4%	84.2%
31	Metronidazole suspension	200mg/5 mL	syrup	POM	1.45	1.31	54.6%	79.0%
32	Nalidixic acid	250 mg	Tab	POM	24.13	21.71	9.1%	10.5%
33	Nystatin	100,000 IU/mL	oral drops	P	6.43	4.83	63.6%	79.0%
34	Oral rehydration salts	20.5 g/L	oral solution	GSL	77.20	82.03	63.6%	89.5%
35	Paracetamol	500 mg	Tab	GSL	3.06	2.68	90.9%	100.0%
36	Paracetamol suspension	120mg/5 mL	oral suspension	GSL	1.45	1.16	100.0%	89.5%
37	Phenobarbital	30 mg	Tab	POM	1.61	1.87	45.5%	89.5%
38	Praziquantel	600 mg	Tab	POM	48.25	47.04	36.4%	63.2%
39	Promethazine	25 mg	Tab	P	3.86	1.93	54.6%	89.5%
40	Promethazine hydrochloride	5mg/5 mL	syrup	P	1.64	1.45	45.5%	68.4%
41	Salbutamol	4 mg	Tab	P	1.61	1.28	45.5%	94.7%
42	Salbutamol suspension	2mg/5 mL	syrup	P	1.06	0.97	54.6%	84.2%
43	Zinc sulphate dispersible	20 mg	Tab	P	4.83	3.86	63.6%	84.2%

### Affordability of essential pediatric medicines in Malawi

**FIGURE 3:**
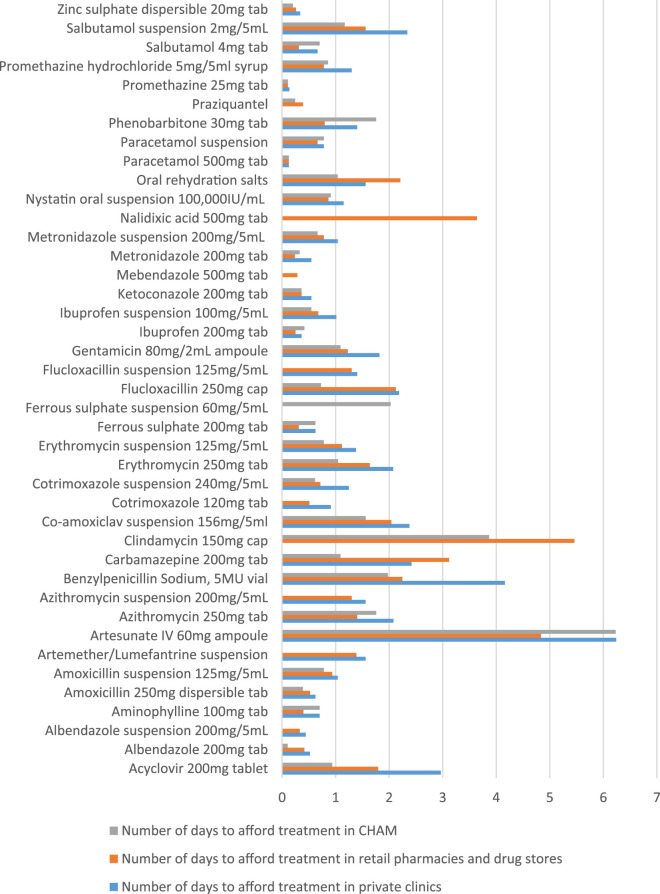
Affordability of pediatric medicines in Malawi.

Affordability was assessed based on various weights as described in the methods section. The selected diseases on which the assessment was done have been attached as supplementary Table 3. Figure 3 provides details of affordability of medicines in which artesunate injection, clindamycin tablet, benzyl penicillin injection and carbamazepine tablet had the highest number of days for a family to afford treatment that required more than 2 days of work. Majority of the formulations were affordable, as they required less than a day to afford the treatment.

Of special note is that all antimicrobials (amoxicillin, cotrimoxazole Metronidazole and erythromycin) that are supposed to be found in primary health facilities were affordable as their number of days to purchase a course of treatment were less than or equal to 1 day in all the sectors. All paracetamol formulations had affordability below 1 day’s wage with the tablet requiring a tiny fraction of a day’s work. The pattern of paracetamol was similar with the non-steroid anti-inflammatory drugs (NSAIDs) ibuprofen, Figure 3 (and supplementary Table 3)

## Discussion

Our study has shown poor availability of pediatric medicines across all major sectors in Malawi. No sector had an overall availability of more than 55% for the targeted medicines that is way below the WHO benchmark of 80%. A sub-analysis of medicines that were supposed to be found in the primary health facilities did not show any improvement towards the WHO benchmark of 80% availability in both CHAM and public sectors. The low availability of pediatric formulation in Malawi is similar with findings from other LMIC (e.g., Ethiopia, Zambia, Kenya, Tanzania, *etc.*) (Chomba et al., 2010; Sado and Sufa, 2016; Abrha et al., 2018; Tadesse et al., 2021). Of major worry is the very low availability of essential antibiotic syrups (amoxicillin, cotrimoxazole and azithromycin) in public sectors. This is based on the fact that Malawi is heavily burdened with infectious diseases, especially the pediatric population (Iturriza-Gómara et al., 2019; Tizifa et al., 2021). This forces the care givers as well as facilities to break adult dosage forms in trying to administer to the young ones. This tendency results in inaccurate dosing in the younger population, for which its effect on treatment outcomes is unknown. The possible cause of lower availability of pediatric dosage forms could be due to lack (or inadequate) of policy guiding the availability of pediatric formulations in Malawi as evidenced by high availability of tablet formulation against syrups/suspensions in this study.

Availability of pediatric medicines in the public sector was low, especially for medicines that were supposed to be found at secondary level. This is not surprising as the medicines are not supposed to be found in primary facilities unless if the facility has a higher qualified clinician (Ministry-of-Health, 2023). The lower availability of pediatric medicines is similar with previous studies that focused on adult medicines in Malawi. Few Studies assessing availability, affordability and prices of essential medicines conducted in Malawi mostly focused on adults with little data available for children (Khuluza and Heide, 2017; Khuluza and Haefele-Abah, 2019). Even though this study focused on pediatric medicines, it still shows that the availability of government-funded medicines in Malawi remains a challenge, 4 years post previous study (Khuluza and Haefele-Abah, 2019).

A sub-analysis of unclassified medicines showed poor availability in the public and CHAM facilities. This is not surprising as these medicines are not supposed to be found due to their categorization. However, a closer look showed that cotrimoxazole 120 mg tablet and nystatin oral drops had a 75% availability in the public sector. This could be attributed to following the Integrated Management of Child Illness (IMCI) guidelines by facilities. Malawi has IMCI unit within the Ministry of Health that is tasked in following up the management of child illness. However, there is a need to incorporate these guidelines in the Malawi Essential Medicine Lists. This would help to properly classify them, and avoid misclassification.

The availability of POM medicines in Drug stores is a worrying trend to the country. This is because drug stores are not supposed to be keeping those medicines based on PMRA’s Act and regulations (Government of Malawi, 2019). The availability of POM medicines in drug stores is a worrying trend in Malawi and Africa where rampant selling of POM medicines is done without authorized prescription from prescriber. More worrying is that most drug stores end up selling the POM medicines without prescription as they are not allowed to handle them (Foster and Bandawe, 2014; Auta et al., 2019). Our results cements information on selling of antibiotic without prescription that has been observed in various countries including in Malawi (Belachew et al., 2021a; Belachew et al., 2021b; Ndaki et al., 2021; Chen et al., 2023). The availability of the antibiotics in the drug stores would in most cases promote antimicrobial resistance due to dispensing without prescription.

Regarding median prices, private clinics were the highest than the rest of the private sector. However, the high prices in private clinics is similar with previous findings within Malawi (Khuluza and Heide, 2017). The median prices observed in this study were higher than the 2017 and 2019 study within Malawi (Khuluza and Heide, 2017; Khuluza and Haefele-Abah, 2019). This could be explained by the differences in years of data collection. However, we did not compare our median prices with the WHO/HAI medicine price indicator due to non-updated of WHO/HAI prices for the past 9 years (World Health Organization, 2023b). The WHO/HAI methodology advised the use of nationally approved prices as a proxy in assessing median price ratio. However, we did not use this method as pricing in Malawi’s pharmaceutical sector has been volatile due to foreign exchange challenges being experienced (World Bank, 2023; Aljezeera, 2023) that has resulted in frequent devaluation of the local currency.

In terms of affordability of essential medicines, we observed a very good affordability of pediatric medicines in Malawi. Almost all pediatric medicines were affordable except for a few products. This is similar with various studies in African countries where the cost of purchasing a course of treatment was less than 1 day’s budget, same with the results of this study (Mahadeo et al., 2022). This is good news for Malawi as the lower prices meant more access of essential pediatric medicines even when the public sector does not have.

Even though most medicines were affordable, the high cost that was needed to purchase full dosages of nalidixic acid, co-amoxiclav, benzyl penicillin and artesunate poses a worry to the pediatric population. More worrying is that these (co-amoxiclav, benzyl penicillin and nalidixic acid) are used as first-line treatment for most microbial infections in Malawi. As for artesunate injection, it is the second line treatment for malarial before referral to secondary hospital and its unaffordability poses challenges to the rural masses. Most CHAM facilities are located in the rural areas (https://cham.org.mw/) of which most the population surrounding the facilities are not working. Therefore there is a need for measures to ensure affordability of these medicines in these sector.

## Limitations

In Malawi, originator brands are not commonly found, as such this was omitted. Median price ratios to the international reference prices were not calculated due to non-availability of the current MSH’s International Medical products Price Guide.

## Conclusion

In the current study, we found poor availability of pediatric formulation among public, faith-based, and private sectors in Malawi. This indicates that there is no improvement in the supply of medicines in Malawi as the results are consistent with the findings of the previous studies. Poor availability of pediatric formulations may affect the quality of care among children and therefore contribute to high morbidity and mortality in Malawi. In order to achieve universal health coverage, the supply of medicines and health commodities needs to consider needs of special populations such as children. (World Bank, 2022; Health_International_Action, 2020).

## Data Availability

The original contributions presented in the study are included in the article/Supplementary Material, further inquiries can be directed to the corresponding author.

## References

[B1] Abrha S. Tadesse E. Atey T. M. Molla F. Melkam W. Masresha B. (2018). Availability and affordability of priority life-saving medicines for under-five children in health facilities of Tigray region, northern Ethiopia. BMC Pregnancy Childbirth 2, 464–469. 10.1186/s12884-018-2109-2 PMC6267819 30497441

[B2] Aljezeera. 2023, ‘Very thin budget’: forex shortage triggers cost-of-living crisis in Malawi. https://www.aljazeera.com/features/2023/10/18/very-thin-budget-forex-shortage-triggers-cost-of-living-crisis-in-malawi.

[B3] Auta A. Hadi M. A. Oga E. Adewuyi E. O. Abdu-Aguye S. N. Adeloye D. (2019). Global access to antibiotics without prescription in community pharmacies: a systematic review and meta-analysis. J. Infect. 78, 8–18. 10.1016/j.jinf.2018.07.001 29981773

[B4] Babigumira J. B. Lubinga S. J. Jenny A. M. Larsen-Cooper E. Crawford J. Matemba C. (2017). Impact of pharmacy worker training and deployment on access to essential medicines for children under five in Malawi: a cluster quasi-experimental evaluation. BMC Health Serv. Res. 17, 638–639. 10.1186/s12913-017-2530-7 28893243 PMC5594492

[B5] Bazargani Y. T. Ewen M. De Boer A. Leufkens H. G. M. Mantel-Teeuwisse A. K. (2014). Essential medicines are more available than other medicines around the globe. PLoS One 9, e87576–e87577. 10.1371/journal.pone.0087576 24533058 PMC3922716

[B6] Belachew S. A. Hall L. Erku D. A. Selvey L. A. (2021a). No prescription? No problem: drivers of non-prescribed sale of antibiotics among community drug retail outlets in low and middle income countries: a systematic review of qualitative studies. BMC Public Health 21, 1056–1113. 10.1186/s12889-021-11163-3 34082726 PMC8173982

[B7] Belachew S. A. Hall L. Selvey L. A. (2021b). Non-prescription dispensing of antibiotic agents among community drug retail outlets in Sub-Saharan African countries: a systematic review and meta-analysis. Antimicrob. Resist Infect. Control 10, 13–15. 10.1186/s13756-020-00880-w 33446266 PMC7807893

[B8] Bigdeli M. Jacobs B. Tomson G. Laing R. Ghaffar A. Dujardin B. (2013). Access to medicines from a health system perspective. Health Policy Plan. 28, 692–704. 10.1093/heapol/czs108 23174879 PMC3794462

[B9] Chen J. Xie Y. Sun Y. Zang R. Dan L. (2023). Sales of antibiotics without a prescription in pharmacies, 2017 and 2021, China. Bull. World Health Organ 101, 317–325A. 10.2471/BLT.22.289435 37131940 PMC10140688

[B10] Chomba E. N. Haworth A. Mbewe E. Atadzhanov M. Ndubani P. Kansembe H. (2010). The current availability of antiepileptic drugs in Zambia: implications for the ILAE/WHO ‘out of the shadows’ campaign. Am. J. Trop. Med. Hyg. 83, 571–574. 10.4269/ajtmh.2010.10-0100 20810822 PMC2929053

[B11] Droti B. O’Neill K. P. Mathai M. Yao Tsidi Dovlo D. Robertson J. (2019). Poor availability of essential medicines for women and children threatens progress towards Sustainable Development Goal 3 in Africa. BMJ Glob. Heal 4, e001306. 10.1136/bmjgh-2018-001306 PMC6797404 31673436

[B12] Exemplars_in_Global_Health (2023). Under-five mortality . https://www.exemplars.health/topics/under-five-mortality (Accessed November 6, 2023).

[B13] Fincham J. E. (2008). Response rates and responsiveness for surveys, standards, and the Journal. Am. J. Pharm. Educ. 72, 43. 10.5688/aj720243 18483608 PMC2384218

[B14] Foster E. K. Bandawe C. R. (2014). How much do patients in Blantyre, Malawi know about antibiotics ana other prescription only medicines? Malawi Med. J. 26, 12–15. 24959319 PMC4062778

[B15] Gitanjali B. (2011). Essential medicines for children: should we focus on a priority list of medicines for the present? J. Pharmacol. Pharmacother. 87, 570–571. 10.4103/0976-500X.77073 PMC3117561 21701637

[B16] Government of Malawi (2019). The pharmacy and medicines regulatory authority Act. https://pmra.mw/the-act/.

[B17] Health_International_Action (2020). Collecting evidence on medicine prices and availability . https://haiweb.org/what-we-do/price-availability-affordability/collecting-evidence-on-medicine-prices-availability/ (Accessed November 13, 2023).

[B18] Hoppu K. Sri Ranganathan S. (2015). Essential medicines for children. Arch. Dis. Child. 100, S38–S42. 10.1136/archdischild-2013-305705 25613966

[B19] IHMA (2023). Child health . https://www.healthdata.org/research-analysis/health-risks-issues/child-health (Accessed November 13, 2023).

[B20] Iturriza-Gómara M. Jere K. C. Hungerford D. Bar-Zeev N. Shioda K. Kanjerwa O. (2019). Etiology of diarrhea among hospitalized children in Blantyre, Malawi, following rotavirus vaccine introduction: a case-control study. J. Infect. Dis. 220, 213–218. 10.1093/infdis/jiz084 30816414 PMC6581894

[B21] Karlsson O. Kim R. Hasman A. Subramanian S. V. (2022). Age distribution of all-cause mortality among children younger than 5 Years in low- and middle-income countries. JAMA Netw. Open 5, e2212692. Epub ahead of print 2022. 10.1001/jamanetworkopen.2022.12692 35587349 PMC9121187

[B22] Khuluza F. Haefele-Abah C. (2019). The availability, prices and affordability of essential medicines in Malawi: a cross-sectional study. PLoS One 14, 02121255–e212222. 10.1371/journal.pone.0212125 PMC6372227 30753219

[B23] Khuluza F. Heide L. (2017). Availability and affordability of antimalarial and antibiotic medicines in Malawi. PLoS One 12, 01753999–e175415. 10.1371/journal.pone.0175399 PMC5395150 28419126

[B24] Mahadeo S. Narain K. Mhlongo L. Chetty D. Masondo L. Zungu M. (2022). The availability of priority medicines for children under 5 years in eThekwini, South Africa. J. Pharm. Policy Pract. 15, 2–7. 10.1186/s40545-021-00402-y 34986904 PMC8728955

[B25] Medicines for Malaria Venture (2013). Focus on Malawi . https://www.theraventrust.org/focus-on-malawi/, 11th Novermber 2023.

[B26] Ministry-of-Health. 2023, Malawi Standard Treatment Guidelines, Incorporating Malawi essential medicines list. Malawi, Africa, 6th Editio.

[B27] Ndaki P. M. Mushi M. F. Mwanga J. R. Konje E. T. Ntinginya N. E. Mmbaga B. T. (2021). Dispensing antibiotics without prescription at community pharmacies and accredited drug dispensing outlets in Tanzania: a cross-sectional study. Antibiotics 10, 1025–1115. 10.3390/antibiotics10081025 34439074 PMC8389015

[B28] Ooms G. I. van Oirschot J. de Kant D. van den Ham H. A. Mantel-Teeuwisse A. K. Reed T. (2023). Barriers to accessing internationally controlled essential medicines in sub-saharan Africa: a scoping review. Int. J. Drug Policy 118, 104078. 10.1016/j.drugpo.2023.104078 37276779

[B29] Perehudoff K. (2020). Universal access to essential medicines as part of the right to health: a cross-national comparison of national laws, medicines policies, and health system indicators. Glob. Health Action 13, 1699342. Epub ahead of print 2020. 10.1080/16549716.2019.1699342 33131456 PMC7605313

[B30] Phillips J. A. Kazembe P. N. Nelson E. A. S. Fissher J. A. F. G. E. (2008). A paediatric Handbook for Malawi. https://www.medbox.org/document/a-paediatric-handbook-for-malawi#GO.

[B31] Reserve_Bank_of_Malawi (2023). Exchange rates . https://www.rbm.mw/Statistics/MajorRates/(Accessed April 30, 2023).

[B32] Sado E. Sufa A. (2016). Availability and affordability of essential medicines for children in the Western part of Ethiopia: implication for access. BMC Pediatr. 16, 40–48. 10.1186/s12887-016-0572-3 26979737 PMC4791837

[B33] Tadesse T. Abuye H. Tilahun G. (2021). Availability and affordability of children essential medicines in health facilities of southern nations, nationalities, and people region, Ethiopia: key determinants for access. BMC Public Health 21, 714–812. 10.1186/s12889-021-10745-5 33849513 PMC8045262

[B34] Tizifa T. A. Kabaghe A. N. McCann R. S. Nkhono W. Mtengula S. Takken W. (2021). Incidence of clinical malaria, acute respiratory illness, and diarrhoea in children in southern Malawi: a prospective cohort study. Malar. J. 20, 473–512. 10.1186/s12936-021-04013-5 34930300 PMC8685799

[B35] Toroitich A. M. Dunford L. Armitage R. Tanna S. (2022). Patients access to medicines – a critical review of the healthcare system in Kenya. Risk Manag. Healthc. Policy 15, 361–374. 10.2147/RMHP.S348816 35256867 PMC8898182

[B36] Unicef (2023). Under-five mortality . https://data.unicef.org/topic/child-survival/under-five-mortality/ (Accessed November 6, 2023).

[B37] Wage_Indicator (2023). Minimum wage – Malawi . https://wageindicator.org/salary/minimum-wage/malawi (Accessed August 24, 2023).

[B38] Watts G. (2007). WHO launches campaign to make drugs safer for children. BMJ 335, 1227. 10.1136/bmj.39423.581042.DB PMC2137042 18079526

[B39] World Bank (2022). GDP (current US$) - South Africa . https://data.worldbank.org/indicator/NY.GDP.MKTP.CD?locations=ZA (Accessed November 12, 2023).

[B40] World Bank (2023). New Malawi economic update calls for urgent action to address macroeconomic imbalances and increase energy access . https://www.worldbank.org/en/news/press-release/2023/07/19/new-afe-malawi-economic-update-calls-for-urgent-action-to-address-macroeconomic-imbalances-and-increase-energy-access (Accessed November 15, 2023).

[B41] World Health Organization (2013). Global Action Plan for the prevention and control of non-communicable diseases 2013-2020. Glob. Action Nucl. Test. Ban. Dipl. End. Cold War., 1–232.

[B42] World Health Organization (2017). Ten years in public health 2007-2017. https://iris.who.int/bitstream/handle/10665/255355/9789241512442-eng.pdf?sequence=1.

[B43] World Health Organization (2021). WHO model list of essential medicines - 22nd list, 2021. Tech. Doc. 2021.

[B44] World Health Organization (2023a). Child mortality (under 5 Years) . https://www.who.int/news-room/fact-sheets/detail/levels-and-trends-in-child-under-5-mortality-in-2020 (Accessed November 7, 2023).

[B45] World Health Organization (2023b). WHO/Health Action International project on medicine prices and availability . https://www.who.int/teams/health-product-and-policy-standards/medicines-selection-ip-and-affordability/who-hai-project-medicine-prices-and-availability (Accessed November 15, 2023).

[B46] World Health Organization. Measuring medicine prices,availability,affordability and price components. Soc. Nat. Resour. 2. 2012, Epub ahead of print 2008. 10.1080/08941920701456422

[B47] Yenet A. Nibret G. Tegegne B. A. (2023). Challenges to the availability and affordability of essential medicines in african countries: a scoping review. Clin. Outcomes Res. 15, 443–458. 10.2147/CEOR.S413546 PMC10276598 37332489

[B48] Yewale V. N. Dharmapalan D. (2012). Promoting appropriate use of drugs in children. Int. J. Pediatr. 2012, 906570–906575. 10.1155/2012/906570 22645620 PMC3356911

