# Supplementary material for: Availability, pricing, and affordability of essential medicines for pediatric population in Malawi
Source: Front Pharmacol. 2024 Apr 11;15:1379250. doi: 10.3389/fphar.2024.1379250 (PMC11043549; doi:10.3389/fphar.2024.1379250)
Supplement: Supplementary file 1 [file Table1.DOCX]

Supplementary table 1

*Supplementary table 1. Pediatric medicines that were studies and justification*

|  | **Disease** | **Name of medicine** | **Strength** | **Dosage form** | **Justification** |
| --- | --- | --- | --- | --- | --- |
| 1 | Antimalarial | artemether /lumefantrine | 20mg/120mg respectively | Dispersible tab | Essential drug on MEML, to be available in all facilities |
|  |  | Artemether lumefantrine | 20mg/5ml + 120mg/5ml | Suspension | Essential drug on MEML, to be available in all facilities |
|  |  | Artesunate-amodiaquine | 25mg/67.5mg, 50mg/135mg & 100mg/270mg | Tab | Essential drug in MEML, Used as second line for treatment of uncomplicated malaria |
| 4 | Antimalarial | Artesunate | 60mg | IV | Essential drug in MEML, used for treatment of severe malaria |
| 5 | Antimalarial | Artesunate | 2 | Antimalarial | Essential drug in MEML, used for treatment of severe malaria |
| 6 | Anthelmintic | mebendazole | 3 | Antimalarial | Essential drug on MEML, be available in all facilities |
| 7 | Anthelminthic | Albendazole | 200mg | Tab | Essential drug in MEML and to be available in all facilities |
| 8 | Anthelminthic | Albendazole | 200mg/5ml | Suspension | Essential drug in MEML and to be available in all facilities |
| 9 | Anti-schistosomal | praziquantel | 600mg | Tab | Essential drug in MEML, used for bilharzia in Malawi |
| 10 | Anti-anaemia | Ferrous sulphate | 200mg | Tab | Essential drug in MEML |
| 11 | Anti-anaemia | Ferrous sulphate | 60mg/5ml | Suspension | Essential drug in MEML |
| 12 | Anti-emetic | promethazine | 25mg | Tab | Essential drug in WHO-HAI global list |
| 13 | Anti-emetic | promethazine | 5mg/ml | Suspension | Essential drug in WHO-HAI global list |
| 14 | Antifungal | Nystatin | 100,000units/ml | Suspension | Essential drug in MEML |
| 15 | Antifungal | ketoconazole | 200mg | Tab | Essential drug in MEML |
| 16 | Antifungal | ketoconazole | 100mg/5ml | Suspension | Essential drug in MEML |
| 17 | Anticonvulsant | phenobarbital | 30mg | Tab | Indicated for use in all health facilities as anticonvulsant in MEML |
| 18 | Anti-herpes | Acyclovir | 200mg | Tab | Essential drug on MEML |
| 19 | Asthma | Salbutamol | 4mg, | Tab | Essential drug in MEML |
| 20 | Asthma | Salbutamol | 2mg/5ml | Suspension | Essential drug in MEML |
| 21 | Asthma | Salbutamol | 0.1mg/dose | Inhaler | Essential drug in MEML |
| 22 | Asthma | aminophylline | 100mg | Tab | Essential drug on MEML |
| 23 | Asthma | aminophylline | 50mg/5ml | Suspension | Essential drug om MEML |
| 24 | CNS | carbamazepine |  | Tab | Used as an anticonvulsant in Malawi |
| 25 | Diarrhea | Zinc sulphate | 20mg | Dispersible tab | Drug is on list of tracer medicines |
| 26 | Diarrhea | Zinc sulphate | 20mg | Non dispersible | Drug is on list of tracer medicines |
| 27 | Diarrhea | Oral rehydration salts |  |  | Essential drug in MEML |
| 28 | Infectious disease | Gentamicin | 40mg/kg | Iv solution | Drug is on the list of Tracer Medicines for measuring stock-outs by Ministry of Health |
| 29 | Infectious disease | Benzylpenicillin | 60mg/kg | Iv solution | Drug is on the list of Tracer Medicines for measuring stock-outs by Ministry of Health |
| 30 | Infectious disease | Co-amoxiclav | 500mg + 125mg | Tab | Essential drug in WHO-HAI global list of medicines, drug is in pediatric handbook for Malawi |
| 31 | Infectious disease | Co- amoxiclav | 125mg/5ml | Suspension | Essential drug in WHO-HAI global list of medicines, drug is in paediatric handbook for Malawi |
| 32 | Infectious disease | Amoxicillin | 250mg | Tab | Essential drug in MEML |
| 33 | Infectious disease | Amoxicillin | 125mg/5ml | Suspension | Essential drug in MEML |
| 34 | Infectious disease | erythromycin | 250mg | Tab | Essential drug in MEML |
| 35 | Infectious disease | erythromycin | 125mg/5ml | Suspension | Essential drug in MEML |
| 36 | Infectious disease | cotrimoxazole | 280mg | Tab | Essential drug in WHO-HAI global list of medicines |
| 37 | Infectious disease | cotrimoxazole | 48mg/ml | Suspension | Essential drug in WHO-HAI global list of medicines |
| 38 | Infectious disease | flucloxacillin | 250mg | Cap | Essential drug in MEML |
| 39 | Infectious disease | flucloxacillin | 125mg/5ml | Suspension | Essential drug in MEML |
| 40 | Infectious disease | Nalidixic acid | 250mg | Tab | Essential drug in WHO-HAI global list of medicines |
| 41 | Infectious disease | azithromycin | 250mg | Tab | Essential drug in MEML |
| 42 | Infectious disease | azithromycin | 200mg/ml | Suspension | Essential drug in MEML |
| 43 | Infectious disease | metronidazole | 200mg | Tab | Essential drug in MEML |
| 44 | Infectious disease | metronidazole | 200mg/5ml | Suspension | Essential drug in MEML |
| 45 | Infectious disease | clindamycin | 150mg | Cap | Essential drug in MEML, drug is in pediatric handbook of Malawi |
| 46 | Infectious disease | clindamycin | 75mg/5ml | Suspension | Essential drug in MEML, drug is in pediatric handbook of Malawi |
| 47 | Pain / antipyretic | paracetamol | 500mg | Tab | Indicated for use in all health facilities in MEML |
| 48 | Pain/inflammation | paracetamol | 125mg/5ml | Suspension | Indicated for use in all health facilities in MEML |
| 49 | Pain / inflammation | Ibuprofen | 200mg | Tab | Essential drug in MEML |
| 50 | Pain/ inflammation | Ibuprofen | 125mg/5ml | Suspension | Essential drug in MEML |
