# Supplementary material for: Availability, pricing, and affordability of essential medicines for pediatric population in Malawi
Source: Front Pharmacol. 2024 Apr 11;15:1379250. doi: 10.3389/fphar.2024.1379250 (PMC11043549; doi:10.3389/fphar.2024.1379250)
Supplement: Supplementary file 2 [file Table2.DOCX]

# S2: DATA COLLECTION FORM

Date:

Name of town/village/district: Name of facility:

Facility Type/Level (Tick one):

1. Public sector facility ☐ (specify level below):

Tertiary care facility ☐

Secondary care facility☐

Primary care facility ☐

Private sector facility ☐

Other (Please specify):

1. Type of price:

Procurement price ☐ Price the patient pays ☐

| A | B | C | D | E | F | G | H | I | J |
| --- | --- | --- | --- | --- | --- | --- | --- | --- | --- |
| **Generic name, dosage form, strength** | **Medicine Type** | **Brand or product name(s)** | **Manufacturer** | **Available yes/no** | **Pack size recommended** | **Pack size found** | **Price of pack found** | **Unit price (4 decimal places)** | **Comments** |
| Artemether/Lumefantrine 20mg+120mg dispersible tab | Highest priced medicine |  |  |  | 30 |  |  | per cap/tab |  |
|  | Most sold generic |  |  |  | 30 |  |  | per cap/tab |  |
|  | Lowest-priced generic |  |  |  | 30 |  |  | per cap/tab |  |
| Artemether/Lumefantrine 20mg/5ml + 120mg/5ml | Highest priced medicine |  |  |  | 60 |  |  | per milliliter |  |
|  | Most sold generic |  |  |  | 60 |  |  | per milliliter |  |
|  | Lowest-priced generic |  |  |  | 60 |  |  | per milliliter |  |
| Artesunate-amodiaquine  25mg/67.5mg, 50mg/135mg & 100mg/270mg | Highest priced medicine |  |  |  | 100 |  |  | per cap/tab |  |
|  | Most sold generic |  |  |  | 100 |  |  | per cap/tab |  |
|  | Lowest-priced generic |  |  |  | 100 |  |  | per cap/tab |  |
| Artesunate 60mg Iv | Highest priced medicine |  |  |  | 60 |  |  | per vial |  |
|  | Most sold generic |  |  |  | 60 |  |  | per vial |  |
|  | Lowest-priced generic |  |  |  | 60 |  |  | per vial |  |
| Artesunate suppository 10mg/kg | Highest priced medicine |  |  |  | 10 |  |  | per bullet |  |
|  | Most sold generic |  |  |  | 10 |  |  | per bullet |  |
|  | Lowest-priced generic |  |  |  | 10 |  |  | per bullet |  |
| Mebendazole 100mg tab | Highest priced medicine |  |  |  | 100 |  |  | per cap/tab |  |
|  | Most sold generic |  |  |  | 100 |  |  | per cap/tab |  |
|  | Lowest-priced generic |  |  |  | 100 |  |  | per cap/tab |  |
| Albendazole 200mg/5ml | Highest priced medicine |  |  |  | 100 |  |  | per milliliter |  |
|  | Most sold generic |  |  |  | 100 |  |  | per milliliter |  |
|  | Lowest-priced generic |  |  |  | 100 |  |  | per milliliter |  |
| Albendazole 200mg tab | Highest priced medicine |  |  |  | 100 |  |  | per cap/tab |  |
|  | Most sold generic |  |  |  | 100 |  |  | per cap/tab |  |
|  | Lowest-priced generic |  |  |  | 100 |  |  | per cap/tab |  |
| Praziquantel 600mg tab | Highest priced medicine |  |  |  | 1000 |  |  | per cap/tab |  |
|  | Most sold generic |  |  |  | 1000 |  |  | per cap/tab |  |
|  | Lowest-priced generic |  |  |  | 1000 |  |  | per cap/tab |  |
| Ferrous sulphate 200mg tab | Highest priced medicine |  |  |  | 1000 |  |  | per cap/tab |  |
|  | Most sold generic |  |  |  | 1000 |  |  | per cap/tab |  |
|  | Lowest-priced generic |  |  |  | 1000 |  |  | per cap/tab |  |
| Ferrous sulphate 60mg/5ml | Highest priced medicine |  |  |  | 100 |  |  | per milliliter |  |
|  | Most sold generic |  |  |  | 100 |  |  | per milliliter |  |
|  | Lowest-priced generic |  |  |  | 100 |  |  | per milliliter |  |
| Promethazine 25mg tab | Highest priced medicine |  |  |  | 1000 |  |  | per cap/tab |  |
|  | Most sold generic |  |  |  | 1000 |  |  | per cap/tab |  |
|  | Lowest-priced generic |  |  |  | 1000 |  |  | per cap/tab |  |
| Promethazine hydrochloride 5mg/5ml | Highest priced medicine |  |  |  | 100 |  |  | per milliliter |  |
|  | Most sold generic |  |  |  | 100 |  |  | per milliliter |  |
|  | Lowest-priced generic |  |  |  | 100 |  |  | per milliliter |  |
| Nystatin 100,000 IU/ml | Highest priced medicine |  |  |  | 20 |  |  | per milliliter |  |
|  | Most sold generic |  |  |  | 20 |  |  | per milliliter |  |
|  | Lowest-priced generic |  |  |  | 20 |  |  | per milliliter |  |
| Ketoconazole 200mg tab | Highest priced medicine |  |  |  | 30 |  |  | per cap/tab |  |
|  | Most sold generic |  |  |  | 30 |  |  | per cap/tab |  |
|  | Lowest-priced generic |  |  |  | 30 |  |  | per cap/tab |  |
| Ketoconazole 100mg/5ml | Highest priced medicine |  |  |  | 100 |  |  | per milliliter |  |
|  | Most sold generic |  |  |  | 100 |  |  | per milliliter |  |
|  | Lowest-priced generic |  |  |  | 100 |  |  | per milliliter |  |
| Phenobarbital30mg tab | Highest priced medicine |  |  |  | 1000 |  |  | per cap/tab |  |
|  | Most sold generic |  |  |  | 1000 |  |  | per cap/tab |  |
|  | Lowest-priced generic |  |  |  | 1000 |  |  | per cap/tab |  |
| Acyclovir 200mg tab | Highest priced medicine |  |  |  | 100 |  |  | per cap/tab |  |
|  | Most sold generic |  |  |  | 100 |  |  | per cap/tab |  |
|  | Lowest-priced generic |  |  |  | 100 |  |  | per cap/tab |  |
| Salbutamol 4mg tab | Highest priced medicine |  |  |  | 1000 |  |  | per cap/tab |  |
|  | Most sold generic |  |  |  | 1000 |  |  | per cap/tab |  |
|  | Lowest-priced generic |  |  |  | 1000 |  |  | per cap/tab |  |
| Salbutamol 2mg/5ml | Highest priced medicine |  |  |  | 100 |  |  | per milliliter |  |
|  | Most sold generic |  |  |  | 100 |  |  | per milliliter |  |
|  | Lowest-priced generic |  |  |  | 100 |  |  | per milliliter |  |
| Salbutamol 0.1mg/dose | Highest priced medicine |  |  |  | 200 |  |  | Per dose |  |
|  | Most sold generic |  |  |  | 200 |  |  | Per dose |  |
|  | Lowest-priced generic |  |  |  | 200 |  |  | Per dose |  |
| Aminophylline 100mg tab | Highest priced medicine |  |  |  | 100 |  |  | per cap/tab |  |
|  | Most sold generic |  |  |  | 100 |  |  | per cap/tab |  |
|  | Lowest-priced generic |  |  |  | 100 |  |  | per cap/tab |  |
| Aminophylline 50mg/5ml | Highest priced medicine |  |  |  | 60 |  |  | per milliliter |  |
|  | Most sold generic |  |  |  | 60 |  |  | per milliliter |  |
|  | Lowest-priced generic |  |  |  | 60 |  |  | per milliliter |  |
| Carbamazepine 200mg tab | Highest priced medicine |  |  |  | 100 |  |  | per cap/tab |  |
|  | Most sold generic |  |  |  | 100 |  |  | per cap/tab |  |
|  | Lowest-priced generic |  |  |  | 100 |  |  | per cap/tab |  |
| Zinc sulphate 20mg dispersible tab | Highest priced medicine |  |  |  | 100 |  |  | per cap/tab |  |
|  | Most sold generic |  |  |  | 100 |  |  | per cap/tab |  |
|  | Lowest-priced generic |  |  |  | 100 |  |  | per cap/tab |  |
| Zinc sulphate 20mg tab | Highest priced medicine |  |  |  | 100 |  |  | per cap/tab |  |
|  | Most sold generic |  |  |  | 100 |  |  | per cap/tab |  |
|  | Lowest-priced generic |  |  |  | 100 |  |  | per cap/tab |  |
| Oral rehydration salts | Highest priced medicine |  |  |  | 200 |  |  | per dose |  |
|  | Most sold generic |  |  |  | 200 |  |  | per dose |  |
|  | Lowest-priced generic |  |  |  | 200 |  |  | per dose |  |
| Gentamicin 80mg/2ml | Highest priced medicine |  |  |  | 30 |  |  | Per ampoule |  |
|  | Most sold generic |  |  |  | 30 |  |  | Per ampoule |  |
|  | Lowest-priced generic |  |  |  | 30 |  |  | Per ampoule |  |
| Benzylpenicillin  5,000,000IU | Highest priced medicine |  |  |  | 100 |  |  | per vial |  |
|  | Most sold generic |  |  |  | 100 |  |  | per vial |  |
|  | Lowest-priced generic |  |  |  | 100 |  |  | per vial |  |
| Co-amoxiclav 625mg tab | Highest priced medicine |  |  |  | 21 |  |  | per cap/tab |  |
|  | Most sold generic |  |  |  | 21 |  |  | per cap/tab |  |
|  | Lowest-priced generic |  |  |  | 21 |  |  | per cap/tab |  |
| Co-amoxiclav 156.25mg/5ml | Highest priced medicine |  |  |  | 100 |  |  | per milliliter |  |
|  | Most sold generic |  |  |  | 100 |  |  | per milliliter |  |
|  | Lowest-priced generic |  |  |  | 100 |  |  | per milliliter |  |
| Amoxicillin 250mg cap | Highest priced medicine |  |  |  | 1000 |  |  | per cap/tab |  |
|  | Most sold generic |  |  |  | 1000 |  |  | per cap/tab |  |
|  | Lowest-priced generic |  |  |  | 1000 |  |  | per cap/tab |  |
| Amoxicillin 125mg/5ml | Highest priced medicine |  |  |  | 100 |  |  | per milliliter |  |
|  | Most sold generic |  |  |  | 100 |  |  | per milliliter |  |
|  | Lowest-priced generic |  |  |  | 100 |  |  | per milliliter |  |
| Erythromycin 250mg tab | Highest priced medicine |  |  |  | 1000 |  |  | per cap/tab |  |
|  | Most sold generic |  |  |  | 1000 |  |  | per cap/tab |  |
|  | Lowest-priced generic |  |  |  | 1000 |  |  | per cap/tab |  |
| Erythromycin 125mg/5ml | Highest priced medicine |  |  |  | 100 |  |  | per milliliter |  |
|  | Most sold generic |  |  |  | 100 |  |  | per milliliter |  |
|  | Lowest-priced generic |  |  |  | 100 |  |  | per milliliter |  |
| Co-trimoxazole 120mg tab | Highest priced medicine |  |  |  | 1000 |  |  | per cap/tab |  |
|  | Most sold generic |  |  |  | 1000 |  |  | per cap/tab |  |
|  | Lowest-priced generic |  |  |  | 1000 |  |  | per cap/tab |  |
| Cotrimoxazole 48mg/ml | Highest priced medicine |  |  |  | 100 |  |  | per milliliter |  |
|  | Most sold generic |  |  |  | 100 |  |  | per milliliter |  |
|  | Lowest-priced generic |  |  |  | 100 |  |  | per milliliter |  |
| Flucloxacillin 250mg cap | Highest priced medicine |  |  |  | 100 |  |  | per cap/tab |  |
|  | Most sold generic |  |  |  | 100 |  |  | per cap/tab |  |
|  | Lowest-priced generic |  |  |  | 100 |  |  | per cap/tab |  |
| Flucloxacillin 125mg/5ml | Highest priced medicine |  |  |  | 100 |  |  | per milliliter |  |
|  | Most sold generic |  |  |  | 100 |  |  | per milliliter |  |
|  | Lowest-priced generic |  |  |  | 100 |  |  | per milliliter |  |
| Nalidixic acid 250mg | Highest priced medicine |  |  |  | 60 |  |  | per cap/tab |  |
|  | Most sold generic |  |  |  | 60 |  |  | per cap/tab |  |
|  | Lowest-priced generic |  |  |  | 60 |  |  | per cap/tab |  |
| Azithromycin 250mg tab | Highest priced medicine |  |  |  | 100/6 |  |  | per cap/tab |  |
|  | Most sold generic |  |  |  | 100/6 |  |  | per cap/tab |  |
|  | Lowest-priced generic |  |  |  | 200/6 |  |  | per cap/tab |  |
| Azithromycin 200mg/ml | Highest priced medicine |  |  |  | 15 |  |  | per milliliter |  |
|  | Most sold generic |  |  |  | 15 |  |  | per milliliter |  |
|  | Lowest-priced generic |  |  |  | 15 |  |  | per cap/tab |  |
| Metronidazole 200mg tab | Highest sold generic |  |  |  | 1000 |  |  | per cap/tab |  |
|  | Most sold generic |  |  |  | 1000 |  |  | per cap/tab |  |
|  | Lowest-priced generic |  |  |  | 1000 |  |  | per cap/tab |  |
| Metronidazole 200mg/5ml | Highest priced medicine |  |  |  | 100 |  |  | per milliliter |  |
|  | Most sold generic |  |  |  | 100 |  |  | per milliliter |  |
|  | Lowest-priced generic |  |  |  | 100 |  |  | per milliliter |  |
| Clindamycin 150mg cap | Highest priced medicine |  |  |  | 24 |  |  | per cap/tab |  |
|  | Most sold generic |  |  |  | 24 |  |  | per cap/tab |  |
|  | Lowest-priced generic |  |  |  | 24 |  |  | per cap/tab |  |
| Clindamycin 75mg/5ml | Highest priced medicine |  |  |  | 100 |  |  | per milliliter |  |
|  | Most sold generic |  |  |  | 100 |  |  | per milliliter |  |
|  | Lowest-priced generic |  |  |  | 100 |  |  | per milliliter |  |
| Paracetamol 500mg tab | Highest priced medicine |  |  |  | 1000 |  |  | per cap/tab |  |
|  | Most sold generic |  |  |  | 1000 |  |  | per cap/tab |  |
|  | Lowest-priced generic |  |  |  | 1000 |  |  | per cap/tab |  |
| Paracetamol 120mg/5ml | Highest priced medicine |  |  |  | 100 |  |  | per milliliter |  |
|  | Most sold generic |  |  |  | 100 |  |  | per milliliter |  |
|  | Lowest-priced generic |  |  |  | 100 |  |  | per milliliter |  |
| Ibuprofen 200mg tab | Highest priced medicine |  |  |  | 1000 |  |  | Per cap/tab |  |
|  | Most sold generic |  |  |  | 1000 |  |  | per cap/tab |  |
|  | Lowest-priced generic |  |  |  | 1000 |  |  | per cap/tab |  |
| Ibuprofen 125mg/5ml | Highest priced medicine |  |  |  | 60 |  |  | per milliliter |  |
|  | Most sold generic |  |  |  | 60 |  |  | per milliliter |  |
|  | Lowest-priced generic |  |  |  | 60 |  |  | per milliliter |  |
