# Supplementary material for: Availability, pricing, and affordability of essential medicines for pediatric population in Malawi
Source: Front Pharmacol. 2024 Apr 11;15:1379250. doi: 10.3389/fphar.2024.1379250 (PMC11043549; doi:10.3389/fphar.2024.1379250)
Supplement: Supplementary file 3 [file Table3.docx]

# Supplementary table 3. Calculation of affordability of essential pediatric medicines

|  |  |  |  |  |  |  | Private clinics | | | Private Pharmacies and drug stores | | | CHAM facilities | | |
| --- | --- | --- | --- | --- | --- | --- | --- | --- | --- | --- | --- | --- | --- | --- | --- |
| No. | Targeted disease | Medicine Name | ATC CODE | Course of Treatment | Number of treat-ment units | Weight of patient used** | Median price in US Cents | Price per course of treat-ment in US $ | Number of days to afford treat-ment | Median price in US Cents | Price per course of treat-ment in US $ | Number of days to afford treat-ment | Median price in US Cents | Price per course of treat-ment in US $ | Number of days to afford treat-ment |
| 1 | Chicken pox | Acyclovir 200mg tablet | J05AB01 | 20 mg per kilogram (kg) of body weight, up to 800 mg, four times a day for five days | 30 | 15-20KG | 18.34 | 5.50 | 2.96 | 11.10 | 3.33 | 1.79 | 5.79 | 1.74 | 0.94 |
| 2 | helminths infection | Albendazole 200mg tab | P02CA03 | 2-tab, 1 day | 2 | 15-20KG | 48.25 | 0.97 | 0.52 | 38.60 | 0.77 | 0.42 | 9.65 | 0.19 | 0.10 |
| 3 | helminths infection | Albendazole suspension 200mg/5mL | P02CA03 | 10ml/day, 1 day | 10 | 15-20KG | 8.20 | 0.82 | 0.44 | 6.13 | 0.61 | 0.33 | 0 | 0 | 0 |
| 4 | Asthma | Aminophylline 100mg tab | R03DA05 | 50mg three times a day, 30 days | 45 | 15-20KG | 2.90 | 1.30 | 0.70 | 1.64 | 0.74 | 0.40 | 2.90 | 1.30 | 0.70 |
| 5 | Upper respiratory tract system-bacterial | Amoxicillin 250mg dispersible tab | J01CA04 | 3 cap/day, 5 days | 15 | 15-20KG | 7.72 | 1.16 | 0.62 | 6.47 | 0.97 | 0.52 | 4.83 | 0.72 | 0.39 |
| 6 | Upper respiratory tract system-bacterial | Amoxicillin suspension 125mg/5mL | J01CA04 | 15ml/day, 5 days | 100 | 15-20KG | 1.93 | 1.93 | 1.04 | 1.74 | 1.74 | 0.94 | 1.45 | 1.45 | 0.78 |
| 7 | uncomplicated Malaria | Artemether /Lumefantrine suspension | P01BF01 | 10ml twice a day x 3 days | 60 | 15-20KG | 4.83 | 2.90 | 1.56 | 4.29 | 2.58 | 1.39 |  |  |  |
| 8 | severe malaria | Artesunate IV 60mg ampoule | P01BE03 | < 20 kg: 3.0 mg/kg at admission, then at 12 hours and 24 hours, then once per day up to 7 days | 8 | 15-20KG | 386.01 | 30.88 | 16.64 | 299.16 | 23.93 | 12.90 | 385.53 | 30.84 | 16.62 |
| 9 | Pneumonia | Azithromycin 250mg tab | J01FA10 | Azithromycin 10mg/kg stat then 5mg/kg q24h for total maximum 5 days | 6 | 15-20KG | 64.37 | 3.86 | 2.08 | 43.43 | 2.61 | 1.40 | 54.39 | 3.26 | 1.76 |
| 10 | pneumonia | Azithromycin suspension 200mg/5mL | J01FA10 | Azithromycin 10mg/kg stat then 5mg/kg q24h for total maximum 5 days | 15 | 15-20KG | 19.30 | 2.90 | 1.56 | 16.08 | 2.41 | 1.30 |  |  |  |
| 11 | Meningitis /pneumonia | Benzylpenicillin Sodium, 5MU vial | J01CE01 | Give Benzylpenicillin 100,000 units/kg q6h, | 4 | 15-20KG | 193.00 | 7.72 | 4.16 | 104.22 | 4.17 | 2.25 | 91.68 | 3.67 | 1.98 |
| 12 | Epilepsy | Carbamazepine 200mg tab | N03AF01 | 20mg/kg per day as maintenance dose for one month | 60 | 15-20KG | 7.48 | 4.49 | 2.42 | 9.65 | 5.79 | 3.12 | 3.38 | 2.03 | 1.09 |
| 13 | Acute osteomyelitis | Clindamycin 150mg cap | J01FF01 | Clindamycin 15-40mgs/kg (4 divided doses) for 7 days | 14 | 15-20KG |  |  |  | 72.38 | 10.13 | 5.46 | 51.27 | 7.18 | 3.87 |
| 14 | Meningitis /pneumonia | Co-amoxiclav 625mg tab | J01CR02 |  |  | 15-20KG | 53.08 | 0 | 0 | 36.33 | 0 | 0 | 40.19 | 0 | 0 |
| 15 | Meningitis /pneumonia | Co-amoxiclav suspension 156mg/5ml | J01CR02 | 1-5 years 5ml of oral suspension (125/31) | 100 | 15-20KG | 4.41 | 4.41 | 2.38 | 3.79 | 3.79 | 2.04 | 2.90 | 2.90 | 1.56 |
| 16 | UTI or pneumonia | Cotrimoxazole 120mg tab | J01EE01 | 10mg/kg every 12 hours for 7 days | 28 | 15-20KG | 6.03 | 1.69 | 0.91 | 3.38 | 0.95 | 0.51 |  |  |  |
| 17 | Upper respiratory tract system-bacterial | Cotrimoxazole suspension 240mg/5mL | J01EE01 | 10mg/kg every 12 hours for 7 days | 100 | 15-20KG | 2.32 | 2.32 | 1.25 | 1.33 | 1.33 | 0.71 | 1.14 | 1.14 | 0.61 |
| 18 | pneumonia | Erythromycin 250mg tab | J01FA01 | 2-8 years: 250 mg 6 hourly for 7 day | 28 | 15-20KG | 13.75 | 3.85 | 2.07 | 10.86 | 3.04 | 1.64 | 6.93 | 1.94 | 1.05 |
| 19 | pneumonia | Erythromycin suspension 125mg/5mL | J01FA01 | 1 month-2 years: 125 mg 6 hourly | 100 | 15-20KG | 2.56 | 2.56 | 1.38 | 2.07 | 2.07 | 1.12 | 1.45 | 1.45 | 0.78 |
| 20 | Anaemia | Ferrous sulphate 200mg tab | B03AD03 | 5-7 years; 80-120 mg q8-12h for 3-6 months | 30 | 15-20KG | 3.86 | 1.16 | 0.62 | 1.93 | 0.58 | 0.31 | 3.86 | 1.16 | 0.62 |
| 21 | Anaemia | Ferrous sulphate suspension 60mg/5mL | B03AA07 | 5-7 years; 80-120 mg q8-12h for 3-6 months | 300 | 15-20KG |  |  |  |  |  |  | 1.25 | 3.76 | 2.03 |
| 22 | Impetigo | Flucloxacillin 250mg cap | J01CF05 | Flucloxacillin 12.5mg/kg body weight 6 hourly for 5-7 | 28 | 15-20KG | 14.48 | 4.05 | 2.18 | 14.09 | 3.94 | 2.13 | 4.83 | 1.35 | 0.73 |
| 23 | Impetigo | Flucloxacillin suspension 125mg/5mL | J01CF05 | Flucloxacillin 12.5mg/kg body weight 6 hourly for 5-7 | 100 | 15-20KG | 2.61 | 2.61 | 1.40 | 2.41 | 2.41 | 1.30 |  |  |  |
| 24 | severe acute malnutrition | Gentamicin 80mg/2mL ampoule | J01GB03 | Gentamycin 7.5mg/kg 24 hourly IV/IM for 7 days | 7 | 15-20KG | 48.25 | 3.38 | 1.82 | 32.57 | 2.28 | 1.23 | 28.95 | 2.03 | 1.09 |
| 25 | mild pain | Ibuprofen 200mg tab | M01AE01 | children 5-10mg/kg {max 40mg/kg/day)_5 days Tx | 20 | 15-20KG | 3.38 | 0.68 | 0.36 | 2.31 | 0.46 | 0.25 | 3.86 | 0.77 | 0.42 |
| 26 | mild pain | Ibuprofen suspension 100mg/5mL | M01AE01 | children 5-10mg/kg {max 40mg/kg/day)_5 days Tx | 100 | 15-20KG | 1.88 | 1.88 | 1.01 | 1.25 | 1.25 | 0.68 | 1.01 | 1.01 | 0.55 |
| 27 | Candidiasis/ oropharyngeal | Ketoconazole 200mg tab | J02AB02 | Children: 1-4 years: Give Ketaconazole 50 mg 12 hourly for 10 - 14 days | 7 | 15-20KG | 14.48 | 1.01 | 0.55 | 9.65 | 0.68 | 0.36 | 9.65 | 0.68 | 0.36 |
| 28 | deworming | Mebendazole 500mg tab | P02CA01 | 100mg EVERY 12HRS for 3 days | 6 | 15-20KG |  |  |  | 8.85 | 0.53 | 0.29 |  |  |  |
| 29 | Mouth ulceration in severe acute malnutrition | Metronidazole 200mg tab | J01XD01 | Metronidazole 7.5 mg/kg 8 hourly for 7 days | 21 | 15-20KG | 4.83 | 1.01 | 0.55 | 2.12 | 0.45 | 0.24 | 2.90 | 0.61 | 0.33 |
| 30 | Mouth ulceration in severe acute malnutrition | Metronidazole suspension 200mg/5mL | J01XD01 | Metronidazole 7.5 mg/kg 8 hourly for 7 days | 100 | 15-20KG | 1.93 | 1.93 | 1.04 | 1.45 | 1.45 | 0.78 | 1.23 | 1.23 | 0.66 |
| 31 | Urinary tract infection | Nalidixic acid 500mg tab | J01MB02 | 55 mg/kg/day orally in 4 equally divided doses for 1 or 2 weeks | 28 | 15-20KG |  |  |  | 24.13 | 6.76 | 3.64 |  |  |  |
| 32 | Mouth sores (candidiasis) | Nystatin oral suspension 100,000IU/mL | D01AA01 | 1 mL twice a day for seven days | 30 | 15-20KG | 7.11 | 2.13 | 1.15 | 5.36 | 1.61 | 0.87 | 5.63 | 1.69 | 0.91 |
| 33 | diarrhoea with some dehydration | Oral rehydration salts | A07CA | Plan B: give 75ml/kg ORS over 4 hours | 5 | 15-20KG | 57.90 | 2.90 | 1.56 | 82.03 | 4.10 | 2.21 | 38.60 | 1.93 | 1.04 |
| 34 | Pain and fever | Paracetamol 500mg tab | N02BE01 | Paracetamol 15mg/kg PO 6 hourly | 8 | 15-20KG | 2.90 | 0.23 | 0.12 | 2.90 | 0.23 | 0.12 | 2.90 | 0.23 | 0.12 |
| 35 | Pain and fever | Paracetamol suspension | N02BE01 | Paracetamol 15mg/kg PO 6 hourly | 100 | 15-20KG | 1.45 | 1.45 | 0.78 | 1.23 | 1.23 | 0.66 | 1.45 | 1.45 | 0.78 |
| 36 | epilepsy (generalized seizures) | Phenobarbitone 30mg tab | N03AA02 | Phenobarbitone 5mg/kg nocte | 90 | 15-20KG | 2.90 | 2.61 | 1.40 | 1.64 | 1.48 | 0.80 | 3.62 | 3.26 | 1.75 |
| 37 | Schistosomiasis | Praziquantel | P02BA01 | Give Praziquantel 40 mg/kg as a single dose | 1.5 | 15-20KG |  |  |  | 48.25 | 0.72 | 0.39 | 30.34 | 0.46 | 0.25 |
| 38 | Urticaria | Promethazine 25mg tab | R06AD02 | Promethazine 6.25-12.5mg for children aged 5-12 yrs for 2 weeks | 7 | 15-20KG | 3.62 | 0.25 | 0.1365 | 2.90 | 0.20 | 0.11 | 2.90 | 0.20 | 0.11 |
| 39 | Urticaria | Promethazine hydrochloride 5mg/5ml syrup | R06AD02 | Promethazine 6.25-12.5mg for children aged 5-12 yrs | 100 | 15-20KG | 2.41 | 2.41 | 1.30 | 1.45 | 1.45 | 0.78 | 1.59 | 1.59 | 0.86 |
| 40 | Asthma | Salbutamol 4mg tab | R03AC02 | 2mg three times a day for one month | 45 | 15-20KG | 2.73 | 1.23 | 0.66 | 1.29 | 0.58 | 0.32 | 2.90 | 1.30 | 0.70 |
| 41 | Asthma | Salbutamol suspension 2mg/5mL | R03AC02 | 2mg three times a day for one month | 300 | 15-20KG | 1.45 | 4.34 | 2.34 | 0.97 | 2.90 | 1.56 | 0.72 | 2.17 | 1.17 |
| 42 | diarrhoea | Zinc sulphate dispersible 20mg tab | A12CB01 | Zinc 10mg PO 24 hourly < 6 months and 20mg if > 6months old for 10 days | 10 | 15-20KG | 6.27 | 0.63 | 0.34 | 4.83 | 0.48 | 0.26 | 3.79 | 0.38 | 0.20 |

** calculation of dosage used the range of a child who weighs between 15 and 20 KG
